# Osmotic Action

**Published:** 1867-05

**Authors:** H. R. Noel

**Affiliations:** Professor of Physiology.


### ARTICLE IV.

*Osmotic Action.* A Lecture delivered February 14, 1867, at the Baltimore College of Dental Surgery. By H. R. Noel, M.D., Professor of Physiology.

Gentlemen :

At our last lecture we completed the review of Digestion. We assigned to each portion of the Digestive Tract its appropriate work—its office—its function ; and a similar disposition was made of the Digestive fluids.

Assuming now that Digestion has been thorough, complete, perfect in all respects and the mass of alimentary material fully prepared ; yet for all practical purposes it is thus far, though within the canal, yet without the system ; for it has not yet entered the circulation, and this it must do before it can be blended with the circulating fluids, or can subserve the purpose of nutrition. The veins—the lacteals, the lymphatics and lymphatic glands, are to receive and work up this crude material into appropriate pabulum. How is this change of position accomplished? By what process is the canal emptied, and the vessels filled? By what subtle force—by what magic power is so rapid and complete a change of base effected?

The act is absorption. The force is Capillary Attraction aided and modified by certain chemico-vital processes.

The whole will be discussed as Osmosis or Osmotic Action.

Certain questions rise for solution. What is Osmosis? What is the theory of Osmotic Action? What are the laws? What are the conditions? What retards and what promotes it?

Lardner in his work upon Natural Philosophy defines Osmosis as "but a manifestation of Capillary Attraction."

Professor Draper says, "The absorbent action of the blood vessels depends upon a force known among physical writers as Capillary Attraction." Other writers give similar definitions.

Professor Dalton thinks "these phenomena depend upon the property belonging to animal membranes of imbibing or absorbing certain fluid substances in a peculiar way."

Do not understand from Prof. Dalton's vague statement, that animal membranes alone, have the power of exhibiting osmotic phenomena. The vegetable world exhibits the same. The inorganic world also has its share.

Osmosis itself does not depend upon vitality. Osmosis, pure and simple, may be shown to depend upon Capillary Attraction, and can be exhibited by "a dead animal membrane"—or by a "porous inorganic body." We shall therefore consider first Capillary Attraction.

With this you are more or less familiar—a few examples will serve to freshen the memory of your chemical and philosophical studies, and bring up the knowledge acquired in the past. The rising of oil in the wick of your lamp,—water in a lump of sugar,—water permeating the walls of some of our houses, are examples.

If you dip a fine glass tube into water and remove it again, a small column of water will remain in the tube, held there against gravity by Capillary Attraction. By experimenting you will find, that the smaller the calibre of the opening in the tube, the higher is the column of retained water ; and we infer at once the stronger the Capillary Attraction. So this force bears some relation to the minuteness of the pores, and seems stronger as the pores are more minute.

Thus far we have a very simple problem, but complexity arises, and new elements are introduced the instant we attempt to make a practical application.

(1). Water rises fast enough in a fine glass tube.

(2). Mercury, though a fluid, *is depressed* below its *hydrostatic level.*

(3). Hydrofluoric acid quietly dissolves the tube itself.

Here we are at once brought to a halt, and conclude that all fluids will not pass through all pores indefinitely ; that the subject is becoming more intricate and new elements enter.

Allow me here to give you three propositions by Prof. Draper, as containing in a condensed form the very information we desire.

I. If the force of attraction of the particles of a solid for those of a liquid be not equal to half the cohesive force of the latter for each other, the liquid will refuse to pass through a pore of that solid substance, and in a capillary tube consisting of it, will be depressed below its hydrostatic level.

Example.—The mercury and glass tube—mercury depressed.

II. If the force of attraction of the particles of a solid for those of a liquid exceeds half of the cohesive force of the latter for each other, but is not equal to the whole force, the liquid will pass through a pore of that solid substance, and in a capillary tube of it, will rise above its hydrostatic level.

Example.—Water and a fine glass tube—water rises in the tube.

III. If the force of attraction of the particles of a solid for those of a liquid exceeds the whole cohesive force of the latter, chemical union between them ensues.

Example.—Action of hydrofluoric acid upon the glass tube.

Here we have expressed certain laws of attraction of particles of fluids and solids, and the phenomena necessarily resulting from such laws; and in the study of osmotic phenomena as exhibited in the animal economy, we shall often have occasion to revert to them.

Remember the facts, as thus far given, have been in reference to a purely physical force; and we must expect some modification, some new phenomena, some new laws and conditions when we apply the force to vitalized structures. Many laws and conditions, many and varied phenomena arise and are grouped under the class of osmotic phenomena, but the combination of physical and vital forces, though mutually modifying, alter not the essential nature of either. As we shall have to deal with osmosis, animal membranes enter as the solid septa, and especially is this true when discussed in relation to Digestion. Moreover we deal with organized structures, vital forces, chemico-vital actions, &c., so that of course the subject at once loses its simplicity, but what it thus loses, is more than compensated by the beauty of the study and the intensity of the interest as we seek the physiological solution of the problem of life, in the blended yet harmonious action of the vital and physical. Lardner gives an example of osmosis and capillary attraction that is well worth our study, well worth a careful analysis, for it is rich in condensed information.

"A glass vessel is filled with alcohol—its surface and rim are securely covered, bound over with an animal membrane, this vessel is then immersed in a vessel of water, and the phenomena watched and explained."

(1). Water passes through the membrane to the alcohol.

(2). Alcohol passes through the membrane to the water.

(3). The water is impregnated with alcohol, and the alcohol is diluted with water—2 currents.

(4). The membrane covering the alcohol, gradually rises dome-like, arching above the vessel.

(5). Remove the glass vessel with its arching membrane, prick the membrane with a needle, and a small jet of water shoots up several feet.

Mark well each step as I repeat it;—remember the five divisions, and we will endeavor to analyze, explain and deduce conclusions.

Before proceeding further, allow me to make one or two other statements, simply a notice of certain facts bearing indirectly upon this experiment. Alcohol though kept in an animal membrane, a bladder for instance, will not penetrate the pores—will not reach the external surface and evaporate; water kept in this way will penetrate, will evaporate from the external surface. Alcohol diluted with water and placed in a like situation, gradually loses its water and acquires strength.

Water and alcohol both pass through the animal membrane; there are two currents—one of water and another of alcohol passing in opposite directions; the water current is the strongest and largest, for the membrane rises dome like over the glass vessel containing the now diluted alcohol, and this could only be caused by a rapid flow of water into the vessel. The jet of fluid rising several feet and the arching membrane, both give evidence of the development of a force. Water is heavier than alcohol, yet the stronger current is from water to alcohol. Water has an affinity for animal membrane, alcohol has not; water will rise in the pores of these membranes; alcohol will not unless they be first filled with water.

Suppose we make divisions and classify the facts, classify our deductions as we analyze and arrange our conclusions under numerical heads.

GENERAL CONCLUSIONS.

(1). Animal membranes have pores through which fluids will pass and therefore they exhibit osmotic action.

(2). Different fluids have different affinities for animal membranes.

(3). In the experiment water had the strongest affinity.

(4). This stronger affinity developes and determines the direction of the stronger current.

(5). Water and alcohol having a mutual affinity for each other, mutually dissolve each other, or at any rate rapidly blend to an equality.

(6). Two fluids having a mutual affinity for each other, and separated by an animal membrane, will pass through and mingle, provided one or both have an affinity also for the membrane.

(7). A fluid in a membrane having no affinity for it, will not fill the pores of the membrane; unless, a second fluid having affinities for both should be upon the opposite side. Draper's first and second laws apply here.

(8). Perhaps in each pore of this membrane, there are two currents, a strong one of water going in; a weak one of alcohol coming out.

Endosmosis would represent the current of water, Exosmosis would represent the current of alcohol, not as regards strength but direction.

(9). The two currents seem to depend upon the fact that each fluid as it reaches the opposite side of the membrane is removed by solution in the other. Also that a current continuous and regular can be produced provided the fluid is removed as fast as it reaches the opposite side of the membrane. Burning of a lamp—evaporation from skin, insensible perspiration, &c., all exemplify the action of this principle.

(10). A strong force is developed, as shown by the arching of the membrane, and also by the jet of fluid when the membrane is pricked. Remember therefore, that in separating fluids by animal membranes, a power may be developed:—and one by no means insignificant.

Affinities of fluids and membranes—currents and direction of currents—the conditions necessary for a current, conditions determining the direction of strongest current, the force developed and conditions of its action, are points to be remembered, as of value in the application of the theory of osmosis to absorption from the intestinal canal. Rigid experimentation—patient and persevering investigation of all the phenomena of life, careful analyses of these phenomena, have determined certain laws and conditions of osmotic action, as observed in the human body.

Headland and Chambers of London, in their admirable works, the one upon "Action of Medicines;" the other upon "The Indigestions," give about the following, as rules or laws as far as the subject is understood at the present time.

*Laws.*

I. Other things being equal the current is stronger from the lighter to the heavier fluid, and the strength of each current bears an increased ratio to its density.

II. Other things being equal—the attraction for the membrane, determines the direction of the stronger current; the fluid having strongest attraction passing rapidly through the membrane.

III. The stronger the affinity between the fluids, the more rapidly they diffuse, therefore the swifter the currents.

IV. The motion of one fluid promotes the flow in the direction of the moving current and the more rapid the current is flowing the more rapidly the osmotic current passes through to join it.

V. Pressure upon one side of the membrane determines the current through to the other; and this is eminently true.

VI. The current is increased from an acid to an alkaline fluid.

The activity of osmosis increases with temperature.

We will now endeavor to explain the practical application of these laws to the subject before us—absorption after digestion ; and bring in such collateral thoughts as may bear upon the discussion.

1. The law of Densities.

The lighter fluid passes more rapidly to the denser—and the rapidity of the currents is inversely proportional to their respective densities. Thus blood being heavier than the contents of the canal after complete digestion, the stronger current would be from the alimentary canal to the vessels. This statement may seem too broad, but is nevertheless true,—the blood is denser, is of greater specific gravity, than the contents of the canal after a thorough digestion. But in certain diseases, as acute anæmia for instance, the reverse may obtain and the blood having lost its solid constituents, may not regain these as rapidly as it does the water, and hence the vessels are filled with a fluid of less specific gravity than normal blood. The normal relation of quantity between the blood and vessels being lost, the vessels are rapidly filled by osmotic action from the fluids contained in surrounding structures, and the blood may regain its normal and natural fluidity by a partial inspissation of the tissues. But here we must at once step in, and assist nature by liquid alimentation, dilution of blood, &c., that the tissues may be compensated, and the blood kept at its normal fluidity.

The intense thirst of the wounded upon the battle-field depends upon these causes, for the blood is diminished in quantity, thickened, and from the whole system rises the demand for water, the solvent—water, the vehicle of conveyance. The vessels may partially fill themselves by osmotic action from the fluids bathing the tissues, but still this is robbing the tissues, and the demand for fluids is urgent and should be promptly met. Caution is necessary, however, or by too prolonged and too free use of very dilute food, a condition simulating chronic anæmia may be induced. In fact you may get the blood at so low a specific gravity that this law may be possibly made to act the other way, and the current be from vessels to the canal. This would be a rare case however. Acute anæmia and chronic anæmia are two very different conditions as regards the system. In chronic anæmia the blood is watery, thin —of low specific gravity, and the tissues saturated with fluids which the lymphatics and various capillaries refuse to take up—for the law of density is reversed; affinity of the fluids changed; attraction of the fluids to the membranes prevented; so here are three laws at least, if no more, annulled for the time being. Solid food is probably best in this case, for we may get two advantages; first, avoid further dilution of the blood; second, by inverting the natural order, cause the too fluid blood to lose a part of its water in the dilution of the food ingested.

We can thus partially inspissate the blood, and the blood in turn may by osmotic action inspissate the tissues to regain its normal amount of water. Thus we obtain a double advantage. Diuretics and drastic carthartics are beneficial in dropsies, from this action, combined with others not bearing directly upon our subject.

*II. Law.*

Attraction for the membrane is a point not so well settled, but we will assume that the digested mass has stronger affinity and the current will be from canal to vessels. But there enters here a strong doubt as to the extent of our knowledge, and a great deal of theory must be admitted There are many substances that change or modify this affinity. Some preparations of iron perversely refuse to pass through the membranes into the vessels, and when exhibited as tonics are carried off in defecation, failing completely to exert any influence whatever upon the blood and tissues. The resinoid elements of aloes, myrrh, colocynth, &c., are probably refused passage through the membrane, and exert a specific influence upon the canal in contact. Different substances have different affinities, and therefore different endosmotic powers; some, as the mineral acids for example, following the third law of Draper, actually corrode, dissolve and destroy the membrane. Some as tannic acid, &c., exert a constringing and condensing effect.

(Conclusion in our next number.)

