# Errata

**Published:** 1867-07

**Authors:** 


In the article on "Osmosis" in May No. 18th page, 25th line read "inversed" instead of "increased."

